# Role of Omega-3 Polyunsaturated Fatty Acids in the Production of Prostaglandin E_2_ and Nitric Oxide during Experimental Murine Paracoccidioidomycosis 

**DOI:** 10.1155/2013/947687

**Published:** 2013-12-25

**Authors:** S. C. Sargi, M. M. O. Dalalio, A. G. Moraes, J. E. L. Visentainer, D. R. Morais, J. V. Visentainer

**Affiliations:** ^1^Department of Agricultural Sciences-Food Science, State University of Maringa, Avenida Colombo 5.790, 87.020-900 Maringá, PR, Brazil; ^2^Department of Basic Health Sciences, State University of Maringa, Avenida Colombo 5.790, 87.020-900 Maringá, PR, Brazil; ^3^Department of Chemistry, State University of Maringa, Avenida Colombo 5.790, 87.020-900 Maringá, PR, Brazil

## Abstract

There has recently been increased interest in the potential health effects of omega-3 polyunsaturated fatty acids on the immune system. Paracoccidioidomycosis is the most important endemic mycosis in Latin America. Macrophages have a fundamental role and act as first line of organism defense. The purpose of this study was to analyze the effect of n-3 fatty acids on the production of PGE_2_ and NO by mice infected with Pb18 and fed a diet enriched with LNA for 8 weeks. To study the effect of omega-3 fatty acids on macrophage activity during experimental paracoccidioidomycosis, mice were infected with Pb18 and fed a diet supplemented with LNA. PGE_2_ in the serum of animals was analyzed and NO in the supernatants of macrophages cultured and challenged *in vitro* with Pb18 was measured. Omega-3 fatty acids seemed to decrease the production of PGE_2_ *in vivo* in the infected group fed an LNA-supplemented diet during the 4th and 8th weeks of the experiment. At the same time, we observed an increase in synthesis of NO by peritoneal macrophages in this group. Omega-3 fatty acids thus appear to have an immunomodulatory effect in paracoccidioidomycosis.

## 1. Introduction

The last few decades have seen increased interest in the potential health effects of omega-3 polyunsaturated fatty acids (n-3 PUFA), especially alpha-linolenic acid (LNA, 18:3n-3), and omega-6 polyunsaturated fatty acids (n-6 PUFA), especially linoleic acid (LA, 18:2n-6) [1]. These are essential fatty acids (EFA) as they are not produced by mammals and must be obtained through the diet [2]. LA is metabolized to arachidonic acid (AA, 20:4n-6) and LNA to eicosapentaenoic acid (EPA, 20:5n-3) and docosahexaenoic acid (22:6n-3), and both are converted by the same sequential desaturation and elongation enzyme systems (Δ6-desaturase, Δ5-desaturase, and elongases) during their biosynthesis. Δ6-desaturase is the enzyme that initiates the cascade of synthesis of AA, EPA, and DHA [3, 4].

EPA and AA are precursors of eicosanoids such as prostaglandins, thromboxanes, and leukotrienes. These are highly bioactive and important mediators in normal physiological and inflammatory as well as immunological processes [5]. AA is the progenitor of two inflammatory mediators, PGE_2_ and LTB_4_, which are produced from the enzymes cyclooxygenase and 5-lipoxygenase, respectively. Whereas EPA is a substrate for the synthesis of PGE_3_ and LTB_5_, these mediators have little inflammatory activity compared to those from AA and compete with AA for these enzymes and thus decrease the production of PGE_2_ and LTB_4_. So, increasing dietary n-3 PUFA can alter the balance of eicosanoids to a slightly inflammatory mixture [6, 7].

Many studies have demonstrated the immunomodulatory effects of n-3 PUFA, but the results are contradictory. Some studies found that n-3 PUFA can suppress T-cell proliferation and proinflammatory cytokine secretion [8]. Other studies found that n-3 PUFA may have anti-inflammatory effects by increasing cytokine secretion and reactive nitrogen species [9].

Paracoccidioidomycosis (PCM) is the most important endemic mycosis in Latin America [10]. The disease is caused by the dimorphic fungus *Paracoccidioides brasiliensis* (Pb) and clinical forms of the disease range from asymptomatic pulmonary lesions to a systemic generalized infection, depending on the balance between fungal virulence and the host response [11, 12]. Many immunological mechanisms are triggered to combat the replication of the fungus, but the main mechanisms involve macrophages, which are the first line of defense against fungal infection [13, 14].

In order to establish the effect of n-3 fatty acids on the immune response against this fungus, the goal of this study was to investigate the action of n-3 fatty acids on the production of PGE_2_ in serum and NO by peritoneal macrophages, from mice infected with Pb18 and fed a diet enriched with LNA for 8 weeks.

## 2. Materials and Methods

### 2.1. Animals and Diet

One hundred and twenty 4-week-old male Swiss mice were provided by the central animal house of the State University of Maringa and were kept in polycarbonate cages. The animals received food and water *ad libitum* and were maintained at 12 h dark/12 h light cycle and a room temperature of 23 ± 1°C. Diets were formulated according to the National Research Council [15], and the experimental diet was nutritionally complete (isocaloric) and was supplemented with 7% of perilla flour. This flour was obtained by grinding perilla seeds that contains 54–64% LNA [16, 17]. The control group received commercial food. The composition of the experimental diet, expressed as g·Kg^−1^, was as follows: wheat bran, 316; corn, 235; soybean meal, 300; perilla flour, 70; premix vitamin and mineral, 45; phosphate dicalcium, 31; and salt, 3. The perilla-enriched diet was pelleted and prepared in bulk, separated into daily portions, vacuum-packed, and stored at 4°C for a maximum of 1 week to prevent fatty acid oxidation. Total lipids and the fatty acid composition of the total lipids of freshly prepared diets were monitored.

### 2.2. Experimental Design

After acclimatization for 7 days, 30 animals were randomly assigned to each of four groups according to the experimental diet: control group uninfected (CGU), control group infected (CGI), perilla group uninfected (PGU), and perilla group infected (PGI). To evaluate the role of n-3 PUFA in PCM, 30 mice from the CGI and PGI groups were inoculated via the intraperitoneal route with 0.2 mL of a fungal suspension of Pb18 at a concentration of 2 × 10^6^ cells/mL. Ten mice from each group were sacrificed on the 1th, 4th, and 8th weeks and peritoneal macrophages were cultured and challenged *in vitro* with Pb18. The levels of PGE_2_ in the serum and NO in the culture supernatant of macrophages and the recovery of viable fungi after coculture were evaluated in duplicate. All experiments were performed according to the guidelines of the Ethics Committee for Animal Research of the State University of Maringa (number 007-2011).

### 2.3. Total Lipids, Fatty Acid Composition, and Quantification of Fatty Acid Methyl Esters in the Diets

Total lipids (TL) in feed were determined according to Bligh and Dyer [18]. Fatty acid methyl esters (FAME) were prepared by methylation of total lipids following Joseph and Ackman [19]. The methyl esters were separated by gas chromatography using a Varian 3300 (USA) gas chromatograph fitted with a flame ionization detector and a fused-silica CP-select CB-Fame capillary column (100 m × 0.25 mm i.d., 0.25 *μ*m cyanopropyl CP-7420) operated at a detector temperature of 240°C and an injection port temperature of 240°C. The column temperature was maintained at 165°C for 12 min and programmed to rise from 165 to 185°C at 40°C/min for 15 min and from 180 to 240°C at 15°C/min in 18 min. The ultrapure gas flows were 1.4 mL·min^−1^ carrier gas (hydrogen), 30 mL·min^−1^ makeup gas (nitrogen), 300 mL·min^−1^ synthetic air, and 30 mL·min^−1^ hydrogen flame gas, split injection, 1 : 100 ratio (injection in triplicate). Retention times and peak area % values were automatically computed by a Varian 4290 integrator. For the identification of FA, FA retention times were compared to those of standard methyl esters (Sigma, USA).

The concentration of FA in mg·g^−1^ of total lipids in feed was measured against tricosanoic acid methyl ester (23 : 0) from Sigma (USA) as an internal standard, as described by Joseph and Ackman [19] and Visentainer [20]. The following formula was used to calculate the concentrations: FA (mg·g^−1^ TL) = (A_X_ × W_IS_ × CF_X_)/(A_IS_ × W_X_ × CF_AE_), where TL is total lipids, A_X_ is the peak area of fatty acids, A_IS_ is the peak area of the internal standard (IS) tricosanoic acid methyl ester (23 : 0), W_IS_ is the weight (mg) of IS added to the sample (in mg), W_X_ is the sample weight (in mg), CF_X_ is the theoretical correction factor, and CF_EA_ is the correction factor of methyl ester for fatty acids.

### 2.4. Fungus

A virulent *P. brasiliensis* strain (Pb18) was used in this study. The yeast cells were cultured in Fava Netto's medium [21] for 7 days at 35°C. For inoculums preparation for infection in mice and peritoneal macrophages and in order to obtain the individual cells, the fungal suspension was homogenized with glass beads in vortex homogenizer (3 cycles of 10 s). Cell viability was determined by differential counting using the vital dye Janus Green; only suspensions with cell viability greater than 90% were used for inoculation.

### 2.5. Isolation, Culture, and Fungicidal Activity of Peritoneal Macrophages

Peritoneal cells were collected from the abdominal cavity of control and infected mice by washing their peritoneal cavities with 3 mL of cold complete RPMI 1640 medium (Sigma Chemical, USA). The cells from each mouse were centrifuged (160 ×g, 10 min), resuspended in RPMI 1640, and adjusted to 2 × 10^6^ cells/mL. Differential cells counts were performed on cell suspensions fixed and stained with 0.05% crystal violet dissolved in 3% acetic acid. For culturing, 100 *μ*L of cell suspension was plated into each well of 96-well flat-bottom tissue culture plates, and cultures were incubated for 2 h at 37°C in 5% CO_2_-95% air_._ Subsequently, nonadherent cells were removed and 100 *μ*L of complete RPMI 1640 containing 10% foetal bovine serum (FBS) (Gibco, Invitrogen) was added to the cellular mixture. The cellular suspension was then incubated for 24 h at 37°C in 5% CO_2_-95% air. Then macrophage cultures were challenged *in vitro* with a fungal suspension (Pb18) at a concentration of 2 × 10^5^ yeast/mL for 18 h. A control culture containing only 100 *μ*L of yeast-form Pb18 was subjected to the same procedures and used for the experimental cultures.

### 2.6. Macrophage Fungicide Activity

After incubation of cocultures at 37°C in 5% CO_2_-95% air for 18 h, the culture supernatants were collected and the wells were washed four times with 250 *μ*L of sterile distilled water to lyse the macrophages and release the intracellular fungus. The corresponding washings were mixed to a final volume of 1 mL. One hundred microlitres of this suspension was placed in Petri dishes and cultured in duplicate with sterile brain-heart infusion agar (Difco, Detroit, MI) supplemented with 4% horse serum, 5% growth factor (culture filtrate of different Pb isolates), and 1% gentamicin. Plates were incubated at 37°C, and the colonies were counted after 7 days. The number of colony-forming units (CFUs) was calculated as the average of the duplicate colony counts.

### 2.7. Macrophage Nitric Oxide Production

Nitric oxide (NO) production was quantified according to the accumulation of nitrite in the culture supernatants of peritoneal macrophages by the Griess reaction as described by Nascimento et al. [22]. Briefly, 50 *μ*L of culture supernatant was incubated with an equal volume of Griess Reagent (1% sulfanilamide, 0.1% naphthylene diamine dihydrochloride, and 2.5% H_3_PO_4_) at room temperature for 10 min. The absorbance at 550 nm was determined using a microplate reader. Conversion of absorbance to *μ*M concentrations of NO was calculated from a standard curve using a known concentration of NaNO_2_ diluted in RPMI 1640 medium. All determinations were performed in quadruplicate.

### 2.8. Determination of Prostaglandin E_2_ Concentration

Blood was collected and serum was then separated by centrifugation at 2500 rpm for 10 min at 4°C. Prostaglandin levels in the serum of animals of all groups (CGU, CGI, PGU, and PGI) were measured by ELISA using the Prostaglandin E_2_ EIA Kit (Cayman Chemicals, Ann Harbor, MI, USA) according to the manufacturer's instructions.

### 2.9. Statistical Analysis

Data were analyzed by Tukey's test after one-way ANOVA using Statistica 8.0 software [23], and the differences were considered significant at *P* < 0.05.

## 3. Results

The total lipid (TL) and fatty acid (FA) composition of the experimental diets is presented in Table 1. The TL values were 6.5% for the control diet and 6.8% for the perilla meal-enriched diet. The quantitative analyses of FA in the diets are expressed in mg of FA per gram of total lipid (mg·g^−1^ TL) and are presented in Table 1. The major FAs in both experimental diets were palmitic acid (16 : 0) and oleic acid (18:1n-9). The control diet had the highest amount of linoleic acid (422.73 mg·g^−1^ TL) and the perilla meal-enriched diet had the highest amounts of alpha-linolenic acid (310.7 mg·g^−1^ TL) (*P* < 0.05).

No significant differences in body weight were observed between the experimental groups at the end of the experiment. The mean body weights were CGU 47.58 ± 4.27 g, CGI 46.47 ± 2.79 g, PGU 46.09 ± 5.2 g, and PGI 48.55 ± 2.28 g.

**Figure 1:**
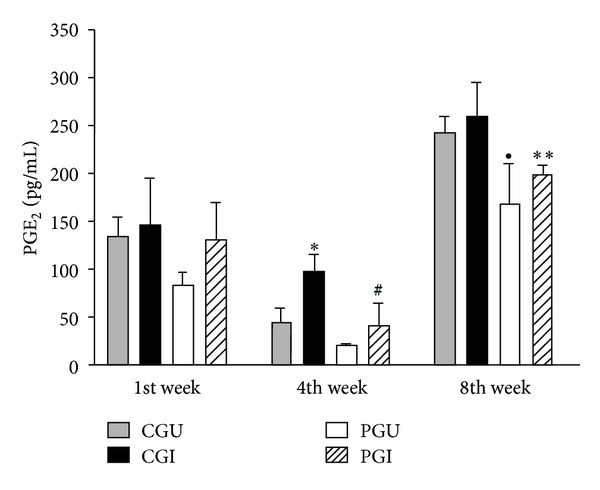
Production of PGE_2_ in serum of animals of all groups during the experiment. Statistically significant differences *P* < 0.05: *CGU versus CGI;  ^#^PGU versus PGI; ^●^CGU versus PGU; **CGI versus PGI.

The effect of PUFA n-3 on the synthesis of prostaglandin E_2_ *in vivo* during murine PCM is shown in Figure 1. In the first week, fatty acid n-3 had no differential effect on the production of PGE_2_ between groups (CGU, CGI, PGU, and PGI). However, during the 4th week there was a significant increase in synthesis in the CGI group compared with the CGU group (*P* = 0.04). An increase in PGE_2_ synthesis by the PGI group (*P* = 0.03) was also observed compared with the PGU group. In the 8th week, synthesis was significantly lower (*P* = 0.04) in the CGU group compared with the PGU group. The pattern was observed in the PGI group, with a decrease (*P* = 0.03) in PGE_2_ production compared with the CGI group (Figure 1).

**Figure 2:**
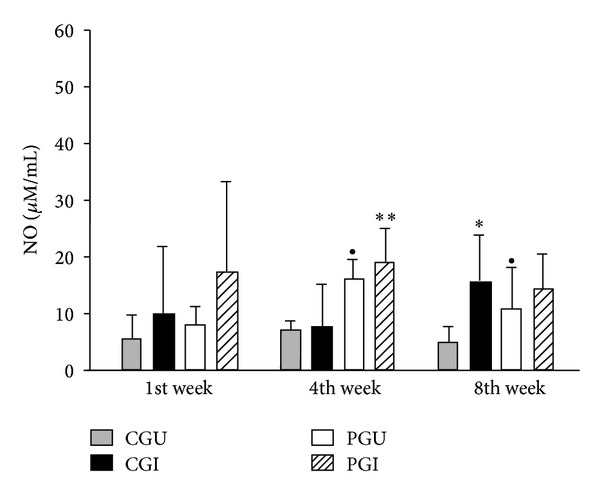
NO generated in culture supernatants of peritoneal macrophages and challenged *in vitro* with Pb18 of animals of all groups during the experiment. Statistically significant differences *P* < 0.05: *CGU versus CGI; ^●^CGU versus PGU; **CGI versus PGI.

The role of the oxidative mechanism and the influence of n-3 fatty acids on this mechanism were assessed by determining NO production in peritoneal macrophages of all groups (CGU, CGI, PGU, and PGI) challenged *in vitro* with Pb18 (Figure 2). During the 1st week of the experiment, NO levels were similar in all groups. However, by the 4th week higher levels (*P* = 0.007) of NO were being produced by macrophages from the PGU group compared with those from the CGU group, when challenged *in vitro*. Likewise the PGI group produced higher NO levels than the CGI group (*P* = 0.0004). During the 8th week there were higher levels (*P* = 0.0005) in the control group CGI compared with CGU. A significant increase (*P* = 0.01) in the production of NO by peritoneal macrophages from PGU compared with CGU was detected (Figure 2). Although not significant, the PGI group had a slightly higher level of NO compared with the PGU group.

**Figure 3:**
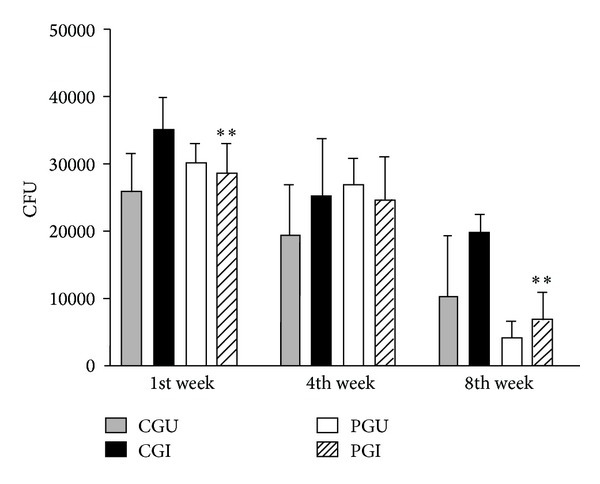
Colony-forming units from culture supernatants of peritoneal macrophages of animals of all groups during the experiment. Statistically significant differences *P* < 0.05: **CGI versus PGI.

Finally, the macrophages fungicide activity was determined through the counting number of CFUs in cultures supernatant (Figure 3). During the 1st week there was a decrease (*P* = 0.02) in the number of CFUs of cultures supernatant in the PGI compared with the CGI group. There were no significant differences in the number of CFUs between experimental groups during the 4th week. During the 8th week, the number of CFUs recovered from cultures of supernatant of peritoneal macrophages from the PGI group was significantly lower than that from the CGI group (*P* = 0.016). The PGU group also showed lower recovery of viable fungi compared with the CGU group, although the difference was not significant (Figure 3). In the control cultures that contained the fungus alone, a CFU above 15000 was observed, indicating that the culture conditions were suitable for the survival of the fungus.

## 4. Discussion

Dietary supplementation with long-chain n-3 PUFA increases the proportion of these fatty acids in immune cells and alters the production of important mediators and regulators of inflammation and immune responses towards a more anti-inflammatory state [24].

The production of eicosanoids during fungal infections plays a critical role in host immune response modulation. Therefore, enhanced prostaglandin production during fungal infection could be a factor in chronic and disseminated disease by suppressing a protective immune response [25]. PGE_2_ is a potent regulator of host immune responses and can inhibit Th1-type immune responses, phagocytosis, and lymphocyte proliferation [26, 27], whereas *Paracoccidioides brasiliensis* can induce the production of these mediators by host cells as an escape mechanism [28].

The findings of the present study demonstrate that n-3 fatty acids did not interfere in PGE_2_ production *in vivo* during the 1st week of the experiment. However, in the 4th week there was a reduction in PGE_2_ synthesis by PGU and PGI compared to CGU and CGI, respectively. The same pattern was observed during the 8th week of the experiment, in the later phase of the disease. These results suggest that n-3 FA can reduce PGE_2_ production [7], a product of AA metabolism. These n-3 fatty acids are also released with AA by phospholipases and act as substrate inhibitors of conversion of AA by cyclooxygenases (COX) [17]. Thus, an increase in consumption of LNA results in an increase in the levels of EPA and DHA cell membrane phospholipids, increasing the synthesis of anti-inflammatory eicosanoids [29, 30], and the incorporation of n-3 PUFA into the membrane of immune cells contributes to determining the severity of the inflammatory process [31].

NO synthesized by macrophages is an important immune mechanism to combat fungal infection. The n-3 fatty acids seemed to influence NO production by peritoneal macrophages from the 4th week of the experiment, when there was an increase in the synthesis of this metabolite by macrophages from the PGU and PGI groups compared to controls. This increase continued until the 8th week. These data suggest that these levels may have been influenced by n-3 PUFA, especially during the later phase of the disease, because eicosanoids, EPA, and DHA can regulate the expression of enzymes that are involved in the production of NO [32, 33]. An increase in NO production also suggests that the host is attempting to defend itself against the fungal infection [14]. NO production by the PGI group was reflected by macrophage fungicide activity since there was a reduced number of CFUs recovered from the culture supernatant during the later phase of the disease. These data suggest that lipids in the diet may affect the microbicidal activity of macrophages.

However, there are many conflicting results about the effect of n-3 fatty acids on the function of macrophages. de la Puerta et al. [34] evaluated the effect of a diet supplemented with 15% fish oil, over 8 weeks, on NO production by murine macrophages stimulated with lipopolysaccharide and observed that NO production was prevented by dietary lipid manipulation. Such results may depend on the experimental models studied, time of feeding, and percentage of FA supplemented.

It seems that supplementation of n-3 fatty acids modified PGE_2_ production* in vivo*, and this alteration was reflected in the *in vitro* production of metabolite NO by macrophages mainly from the fourth to the 8th week of the experiment. Macrophages are the first cells to interact with the fungus and their digestive and killing abilities, along with cytokines secreted in response to this challenge, are crucial to the fate of the infectious process [35]. The capacity for phagocytosis by macrophages is influenced by membrane lipid composition and it is possible that the incorporation of n-3 FA into cell membrane phospholipids influences phagocytosis [36].

## 5. Conclusion

The n-3 fatty acids influenced the production of PGE_2_ and the microbicidal activity of macrophages by increasing the production of NO between the initial and later phases of PCM. How the dietary fatty acids alter eicosanoids and the relationship with macrophage microbicidal activity remains unknown but could be due to an alteration in the levels of the eicosanoid precursor. More studies using LNA should be conducted to elucidate the effect of n-3 and n-6 PUFA on the immune response during experimental murine PCM.

## Figures and Tables

**Table 1:** Total lipid content and fatty acid composition of the experimental diets.

Composition	Control diet	Perilla meal-supplemented diet
	Means ± SD	Means ± SD
Total lipids (%)	6.5 ± 1.15	6.8 ± 1.4

Fatty acid (mg/g total lipids)		
16:0	127.8^a^ ± 0.23	101.8^b^ ± 1.67
17:0	0.7 ± 0.02	nd
18:0	26.5^a^ ± 0.12	16.9^b^ ± 0.26
18:1n-9	211.5^a^ ± 0.69	152.4^b^ ± 2.05
18:1n-7	9.7 ± 0.03	9.5 ± 0.11
18:2n-6 (LA)	422.7^a^ ± 1.75	265.2^b^ ± 1.38
18:3n-6	0.2 ± 0.29	nd
18:3n-3 (LNA)	34.5^a^ ± 0.08	310.7^b^ ± 1.80
20:0	2.9^a^ ± 0.01	1.7^b^ ± 0.01
20:1n-9	3.4^a^ ± 0.02	2.5^b^ ± 0.07

MUFA	222.7^a^ ± 0.36	136.1^b^ ± 1.06
SFA	157.9^a^ ± 0.21	120.3^b^ ± 0.95
PUFA	464.0^a^ ± 0.87	577.3^b^ ± 0.97
n-6	422.9^a^ ± 1.75	266.6^b^ ± 0.99
n-3	34.5^a^ ± 0.08	310.7^b^ ± 1.80
PUFA/SFA	2.9 ± 0.05	8.3 ± 0.02
n-6/n-3	12.2^a^ ± 0.03	0.9^b^ ± 0.05

PUFA: polyunsaturated fatty acid, MUFA: monounsaturated fatty acid, SFA: saturated fatty acid, n-6: omega-6 fatty acid, and n-3: omega-3 fatty acid. Results expressed as mean ± standard deviation for analysis in nine replicates. *n* = 10. Means followed by different letters on the same line are significantly different (*P* < 0.05) by Tukey's test and *t*-test. Control diet versus perilla meal-enriched diets. nd: not detected.
